# Supplementary figures and images for: Hybridogenesis in the Water Frogs from Western Russian Territory: Intrapopulation Variation in Genome Elimination
Source: Genes (Basel). 2021 Feb 8;12(2):244. doi: 10.3390/genes12020244 (PMC7914630; doi:10.3390/genes12020244)

Figure S1

*Sox3*

*Rhodopsin*

**RR**  
(P2-1)

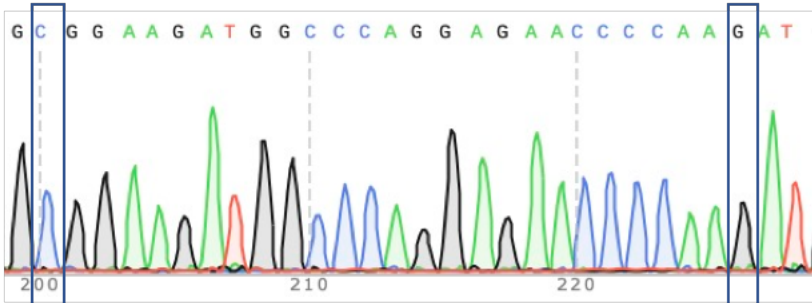

**LL**  
(P1-2)

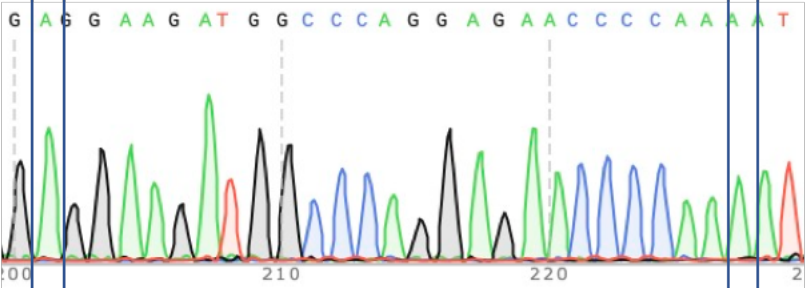

**RL**  
(P1-1)

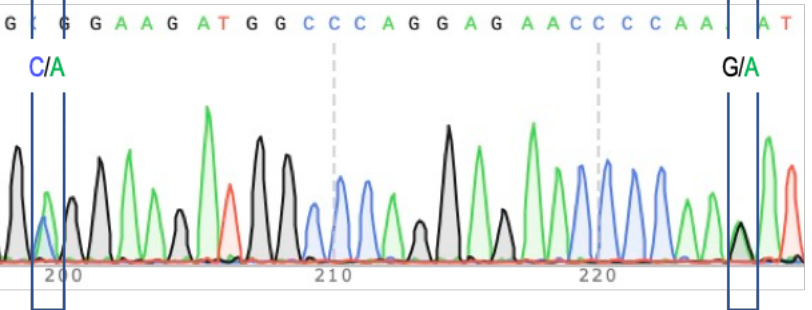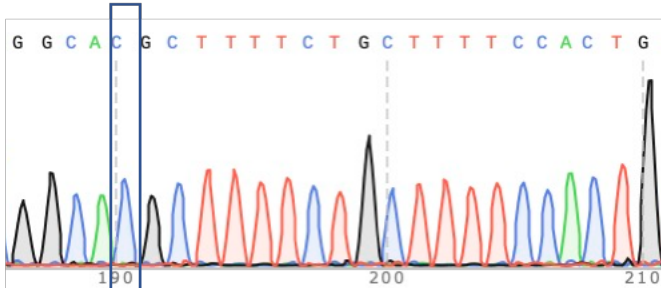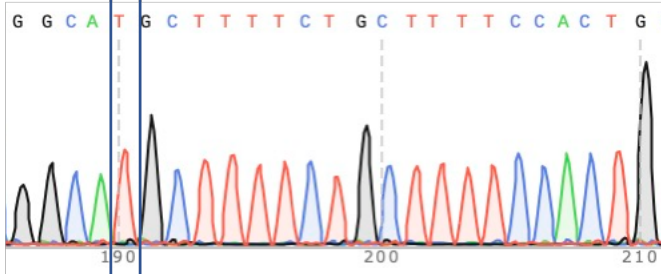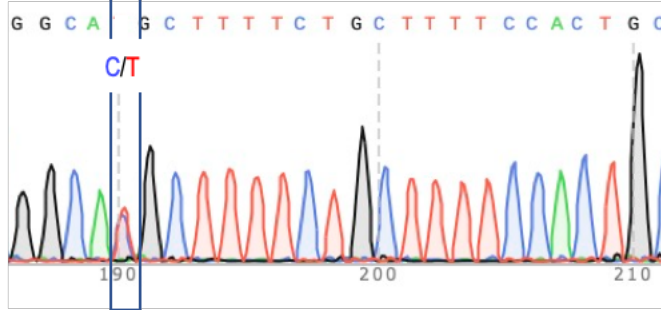

Figure S2

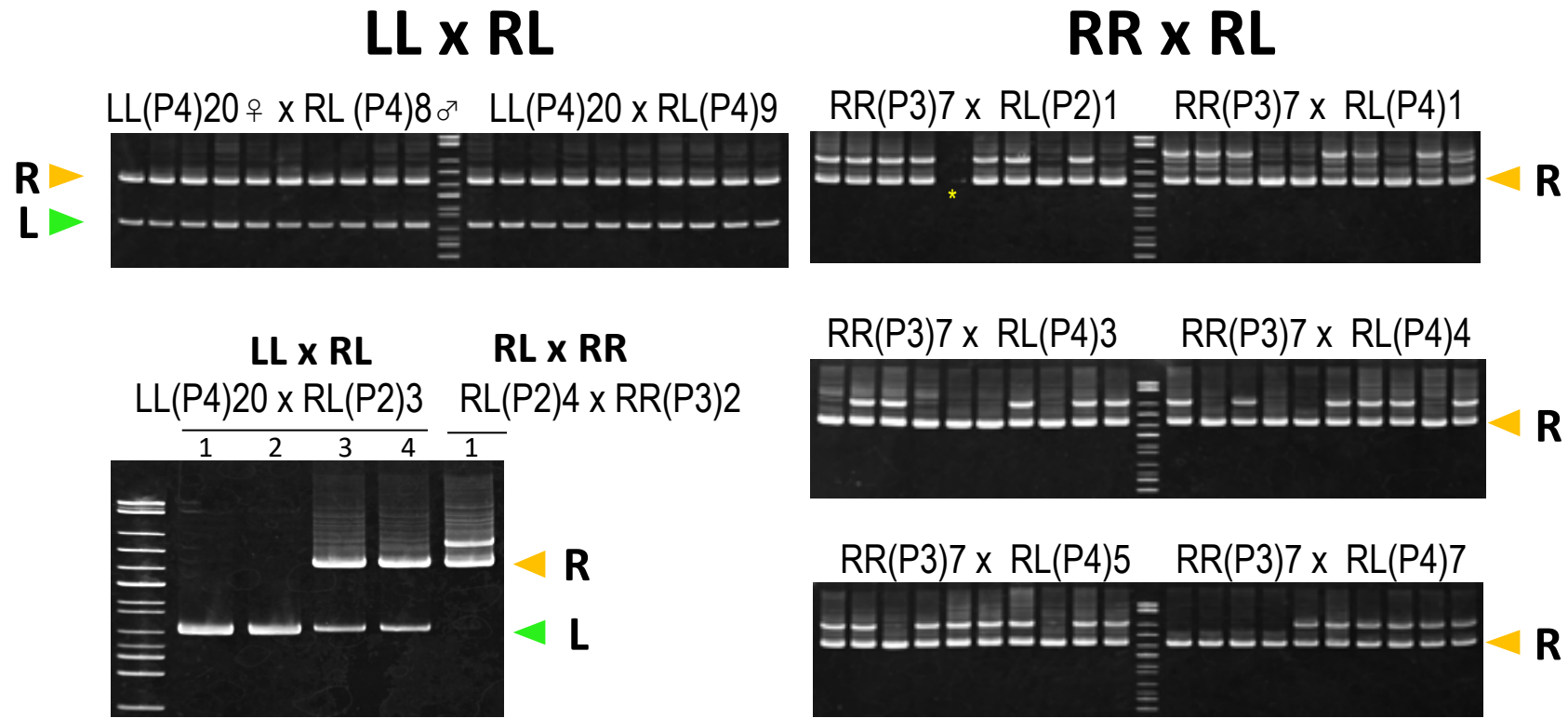

Figure S3

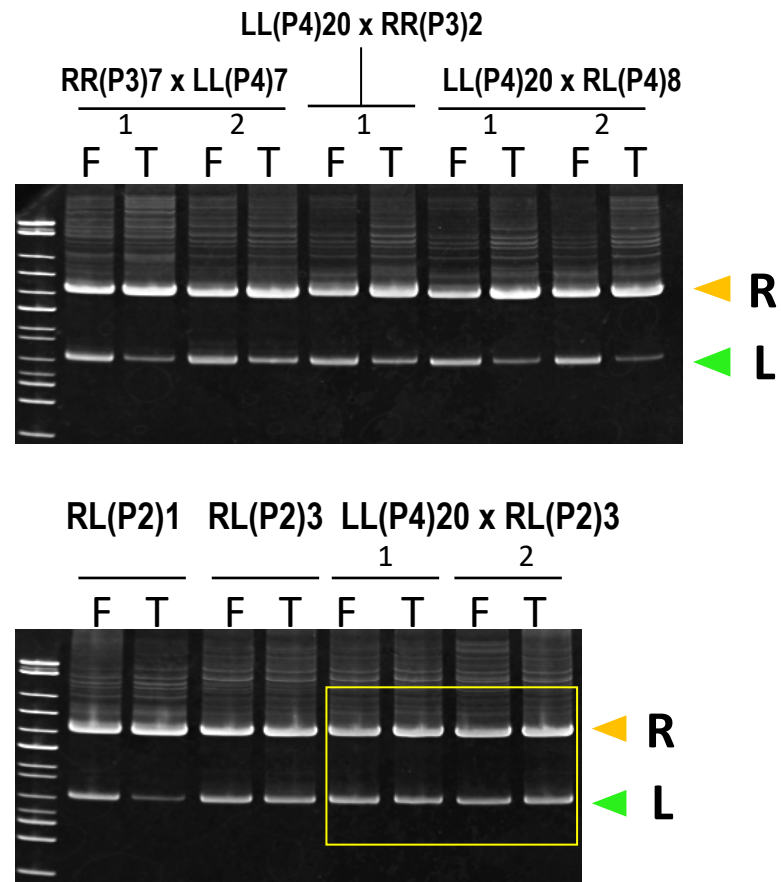

Figure S4

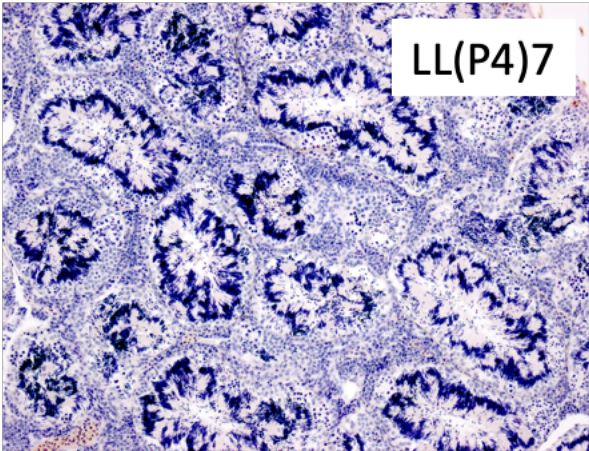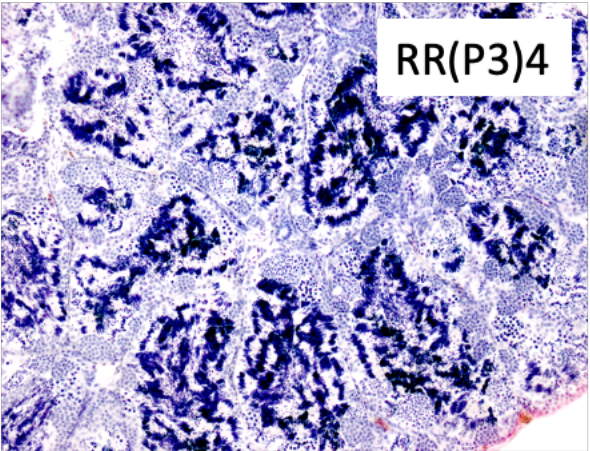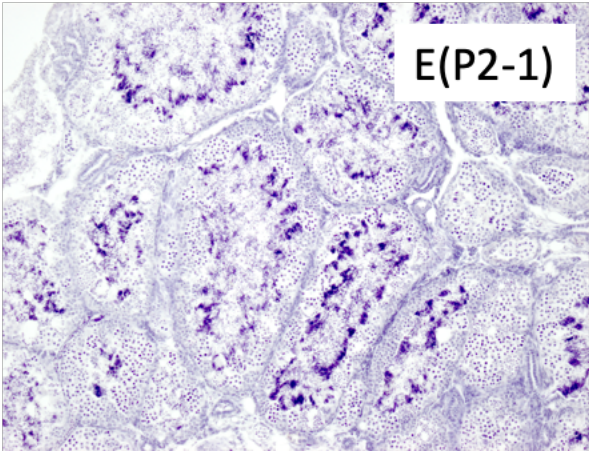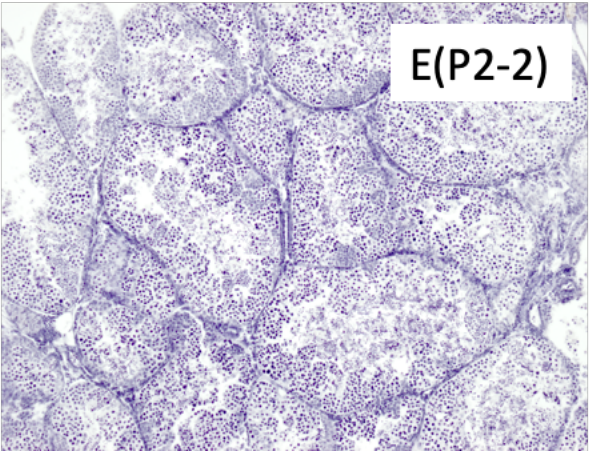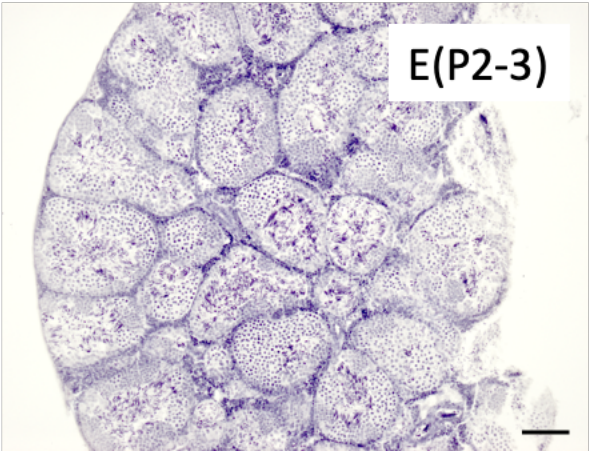

Figure S5

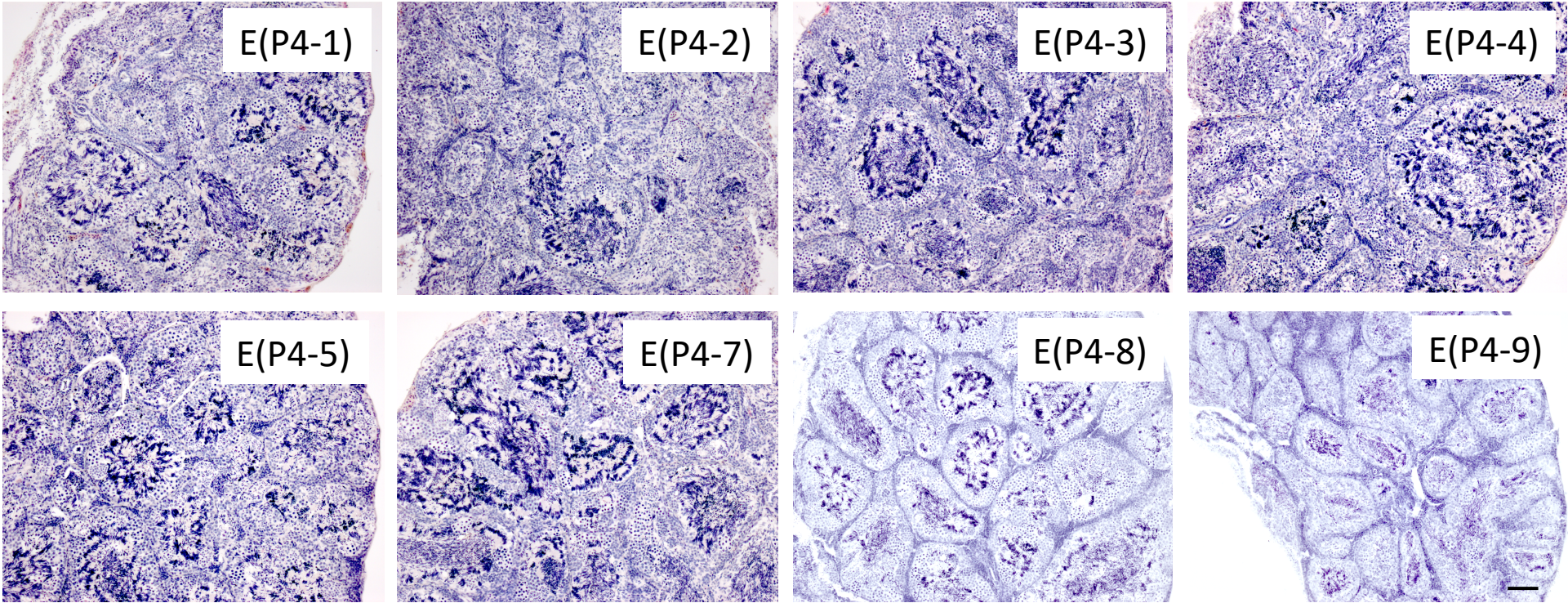

Supplement: Supplementary file 1 [file genes-12-00244-s001.zip › Supplemental figures and tables/Figures S (revised version).pdf]
